# Method to determine the statistical technical variability of SUV metrics

**DOI:** 10.1186/s40658-022-00470-2

**Published:** 2022-06-06

**Authors:** Giulia M. R. De Luca, Jan B. A. Habraken

**Affiliations:** Department of Medical Physics, St. Antonius Hospital, Nieuwegein, The Netherlands

**Keywords:** Standard uptake value, PET quantification, Variation in standard uptake value

## Abstract

**Background:**

The Standardized Uptake Value (SUV) Max, SUVMean, and SUVPeak are metrics used to quantify positron emission tomography (PET) images. In order to assess the significance of a change in these metrics for diagnostic purposes, it is relevant to know their variation. The sources of variation can be biological or technical. In this study, we present a method to determine the statistical technical variation of SUV in PET images.

**Results:**

This method was tested on a NEMA quality phantom with spheres of various diameters with a full-length acquisition time of 150 s per bed position and foreground-to-background activity ratio of F^18^-2-fluoro-2-deoxy-d-glucose (FDG) of 10:1. Our method divides the 150 s acquisition into subsets with statistically independent frames of shorter reconstruction length. SUVMax, Mean and Peak were calculated for each reconstructed image in a subset. The coefficient of variation of SUV within each subset has been used to estimate the expected coefficient of variation at 150 s reconstruction length. We report the largest coefficient of variation of the SUV metrics for the smallest sphere and the smallest variation for the largest sphere. The expected variation at 150 s reconstruction length does not exceed 6% for the smallest sphere and 2% for the largest sphere.

**Conclusions:**

With the presented method, we aim to determine the statistical technical variation of SUV. The method enables the evaluation of the effect of SUV metric choice (Max, Mean, Peak) and lesion size on the technical variation and, therefore, to evaluate its relevance on the total variation of the SUV value between clinical studies.

## Introduction

Positron emission tomography (PET) has become an indispensable diagnostic tool, especially in combination with computed tomography (CT) for attenuation correction. Furthermore, PET-CT imaging provides a combined view of functional and morphological information of the patient.

The radio-ligand F^18^-2-fluoro-2-deoxy-D-glucose (FDG) has ensured the success of PET-imaging. The glucose component of the molecule has a higher uptake in malignant than healthy cells [1], and the F^18^ component provides the detectability in PET-CT systems.

The nuclear medicine physician can evaluate PET-CT images visually. However, an important advantage of PET imaging is that the uptake can be quantified in absolute measures. Quantification of FDG PET enables the staging of cancer and the quantitative comparison of follow-up studies to track the evolution of cancer and the response to tumour therapy [2]. The degree of variability of the quantification methods limits the widespread implementation of quantitative comparison of PET images. Therefore, reliable image quantification requires minimizing inter- and intra-observer variability as much as possible and estimating the measurement errors on the quantification parameters.

One of the metrics for the comparison of F^18^-FDG PET images is the Standardized Uptake Value (SUV). The most used SUV metrics are the SUVMax and the SUVMean. With SUVMax, only the voxel with the highest uptake is considered, while with SUVMean, all the voxels in a certain region or volume are taken into account. SUVMax has a low inter- and intra-observer variability but a high statistical technical variation. SUVMean, on the other hand, has a lower technical variation but a higher inter- and intra-observer variability since the thresholds for the contours of the volume are a determining factor of the result. SUVPeak could be a “best of both worlds” metric because it includes the voxels in a fixed limited volume around the voxel with maximum value. SUVPeak might improve reproducibility for SUV quantification, especially in the most metabolically active tumour regions [3].

The proposed framework for PET Response Criteria in Solid Tumours (PERCIST) suggests considering a 30% change in SUV as a significant variation of tumour activity [4]. Decreasing the variability of SUV quantification will enable the detection of smaller significant changes and therefore enable earlier detection of degeneration or the effect of therapy. Being aware of the expected variation and standardizing the process as much as possible is therefore essential.

In test–retest studies, patients are injected and scanned twice to assess the variation of the complete PET imaging process. However, the knowledge of the components building up the total variation can help to reduce this variation.

Theoretically, the variation in a PET measurement consists of biological variability and technical variability [5]. Biological variability arises from variations in blood glucose, paravenous administration of FDG and FDG uptake. Biological variation can be minimized by standardizing the patient preparation with a protocol defining diet and uptake time. Further, the technical variation also plays a role in the process. Several studies have shown that important factors affecting the technical variation of SUV are variations in image reconstruction and scanner characteristics [6–8]. Standardizing the positioning of the patient, acquisition, reconstruction and quantification protocol, and PET system calibration will reduce the technical variation. However, a certain amount of technical variation is unavoidable due to the statistical variations of the F^18^ decay and its effect on the image reconstruction. This is a “statistical technical variation”, so the part of the technical variation that cannot be minimized by standardization but is intrinsically present on statistical grounds. Knowing the statistical technical variation gives us insights into the origin of the total variation.

Since the statistical technical variation differs between SUV metrics, it is important to be able to quantify it specifically for each metric in use. Furthermore, the statistical technical variability can be scanner- and reconstruction-specific and will be dependent on the size of the lesions. The assessment of the statistical technical variation gives insight into its contribution to the total variation and enables the quantification of the effect of different parameters on the technical variation.

In this study, we present a method to estimate the statistical technical variation of different SUV metrics and lesion sizes of images acquired with the same scanner and reconstructed with the same algorithm. One PET acquisition is divided into subsets of different reconstruction lengths, and the standard deviation between the SUV metrics in the subsets is used to estimate the coefficient of statistical technical variation (from here called coefficient of variation) of the SUV values of the total acquisition. The proposed method determines the coefficient of variation of SUV metrics in order to establish the relevance of this variation in the context of other fluctuations.

The method is described and applied on a 150-s acquisition of a NEMA image quality phantom with a foreground-to-background activity ratio of 10:1 as an example of application. The coefficients of variation of SUVMax, SUVMean and SUVpeak of the different spheres in the phantom are presented and discussed as a function of lesion size. With the presented method, we want to provide a simple method to estimate the statistical technical variability of PET images with the goal of evaluating its impact on the total technical and biological variation between PET images.

## Material and methods

The method that we describe in this paper for estimating the coefficient of variation of SUV metrics between PET images consists of three main steps:

- Acquisition of the original dataset
- Generation and reconstruction of subsets with shorter reconstruction length
- Calculation of SUV coefficient of variation within the subsets, and translation to the coefficient of variation of the original dataset with full reconstruction length.

### Image acquisition and reconstruction

A NEMA NU2–2007 image quality phantom was imaged on a Philips Gemini TF PET/CT system (Philips Healthcare, Andover, MA). PET reconstructions were made using the scanner’s default Ordered Subset Expectation Maximization (OSEM) reconstruction algorithm with 33 subsets, 3 iterations, matrix size of 144 × 144, and voxels of 4 × 4 × 4 mm. No Gaussian filter was applied. The reconstruction was corrected for geometrical response and detector efficiency (normalization), random coincidences, scatter and attenuation. Data were stored in the list mode to be able to reconstruct subsets with different reconstruction lengths. All list-mode reconstructions were decay-corrected to the start time of the acquisition.

The NEMA phantom acquisitions were performed according to the requirements for the EANM/EARL FDG-PET/CT accreditation [9]. The NEMA phantom is composed of a fillable torso compartment acting as background, a cylindrical insert in the centre of the torso compartment and six fillable spheres of different diameters (10 mm, 13 mm, 17 mm, 22 mm, 28 mm and 37 mm) placed around the central insert. The fillable torso compartment and the spheres were filled with a solution of water and F^18^-FDG. At the starting moment of the scan, the activity concentration was 2.10 MBq/ml in the torso background compartment and 20.04 MBq/ml in the spheres, resulting in an actual sphere-to-background ratio of 9.6:1 (the aim was 10:1) [10] .

The original dataset was acquired with a 150-s frame duration. The total acquisition time was 10 min (600 s, 4 bed positions). The full-length 150-s list-mode acquisition was divided into subsets of shorter reconstruction lengths varying from 4 to 30 s. An attenuation-corrected reconstruction was performed for different reconstruction lengths, generating as many images as possible per subset, without using the same coincidences multiple times by varying the starting time of the reconstruction. For example, for the first subset (4 s reconstruction length), the first image was reconstructed using the coincidences recorded between 0 and 4 s, the second image by using the coincidences recorded between 5 and 8 s and so on, varying the starting moment of the reconstruction, generating a total of 37 images. The longest reconstruction length was 30 s, generating a subset of five images. Fourteen subsets were generated with 4 s, 6 s, 8 s, 10 s, 12 s, 15 s, 17 s, 19 s, 20 s, 22 s, 24 s 26 s, 28 s and 30 s reconstruction lengths.

The Philips reconstruction software automatically corrected each reconstruction for the decay of ^18^F (half-life of 109.7 min [11]), compensating for the time difference between the start of the study and the start of the reconstruction by using an opportune scaling factor.

### Image analysis

The datasets were analysed using a Python 3.7.0 script (default, Jun 28 2018, 08:04:48) [MSC v.1912 64 bit (AMD64)]. The algorithm automatically detected the spheres and their central 2D plane. Different SUV metrics were calculated in each image of the subsets:

- SUVMax 2D: the maximum voxel value in the central 2D plane;
- SUVMax 3D: maximum voxel value in each sphere;
- SUVMean 2D: the average value of the voxels in the central 2D plane of each sphere;
- SUVMean 3D: the average value of the voxels in each sphere.

SUVMean 2D and 3D were calculated considering the complete 2D central plane or 3D volume, respectively, without using thresholding techniques based on pixel values or on a percentage of the maximum value.

- SUVPeak: the average value within a 1-cm^3^ sphere centred on the maximum value of the sphere [12]. The algorithm fitted the sphere, found the maximum voxel value in the 3D volume, used this voxel as the centre of a spherical region of interest (ROI) of 1 cm^3^ and calculated the average value within the 1-cm^3^ sphere.

The values of the different SUV metrics were calculated for each image in a subset. The SUV value populations have been tested for normality with a Kolmogorov–Smirnov test, and all subsets matched the characteristics of a normal distribution. The test was run on non-log-transformed data and on log-transformed data. The SUV metrics of the different images in a subset were averaged, and their standard deviation was calculated. The coefficient of variation of the SUV metrics was calculated as the standard deviation divided by their average value multiplied by 100.

We can assume a random sampling model, with no correlations, for independent and identically distributed random measurements. The different subsets did not differ in activity or voxel dimensions, and the quantification of the SUV metrics was done by using the same ROI dimension. We can therefore describe the ratio of the standard deviations (SD) of two independent subsets as:

1 $$\frac{\hbox{SD1}}{\hbox{SD2}}={\left(\frac{\hbox{RL1}}{\hbox{RL2}}\right)}^{-\frac{1}{2}}$$

With SD1 and SD2 being the standard deviations and RL1 and RL2 being the reconstruction lengths [7]. By using the measured coefficient of variation of the SUV in a subset as SD1 and the length of the reconstruction of the specific subset as RL1, it is possible to estimate the coefficient of variation SD2 between repetitions of scans with reconstruction length RL2 according to:

2 $$\hbox{SD2}={\hbox{SD1} \left(\frac{\hbox{RL1}}{\hbox{RL2}}\right)}^\frac{1}{2}$$

Formula 2 is used to calculate the estimated coefficient of variation of the SUV at different reconstruction lengths using the coefficient of variation of the other subsets. In this case, we could calculate (14 − 1 =) 13 estimations of the SD2 for each coefficient of variation.

Formula 2 is also used to calculate the estimated coefficient of variation of SUV metrics at reconstruction length SD2 = 150 s using the coefficient of variation of each subset. We illustrate the procedure for the subset with a reconstruction length of 10 s to estimate the SD2 at a reconstruction length of 150 s. For the reconstruction length of 10 s, we obtained 15 subsets and we could therefore estimate 15 SUV metrics. We then calculated the standard deviation between the 15 SUV metrics and used it as SD1 in Formula 2. The ratio between the reconstruction lengths was also taken into account according to Formula 2 (in this example, it is 10/150). Since we divided our acquisition into 14 different subsets of different reconstruction lengths, we could calculate 14 different estimations of the SD2 of reconstruction length 150 s and evaluate their mean and standard deviation.

Lastly, the population of two adjacent spheres (10 mm and 13 mm, 13 mm and 17 mm, 17 mm and 22 mm, 22 mm and 28 mm, 28 mm and 37 mm) was tested ( two-sample t-test assuming unequal variances with a significance level alpha = 0.05) to verify whether there was a significant difference in SUV between adjacent spheres.

## Results

**Fig. 1:**
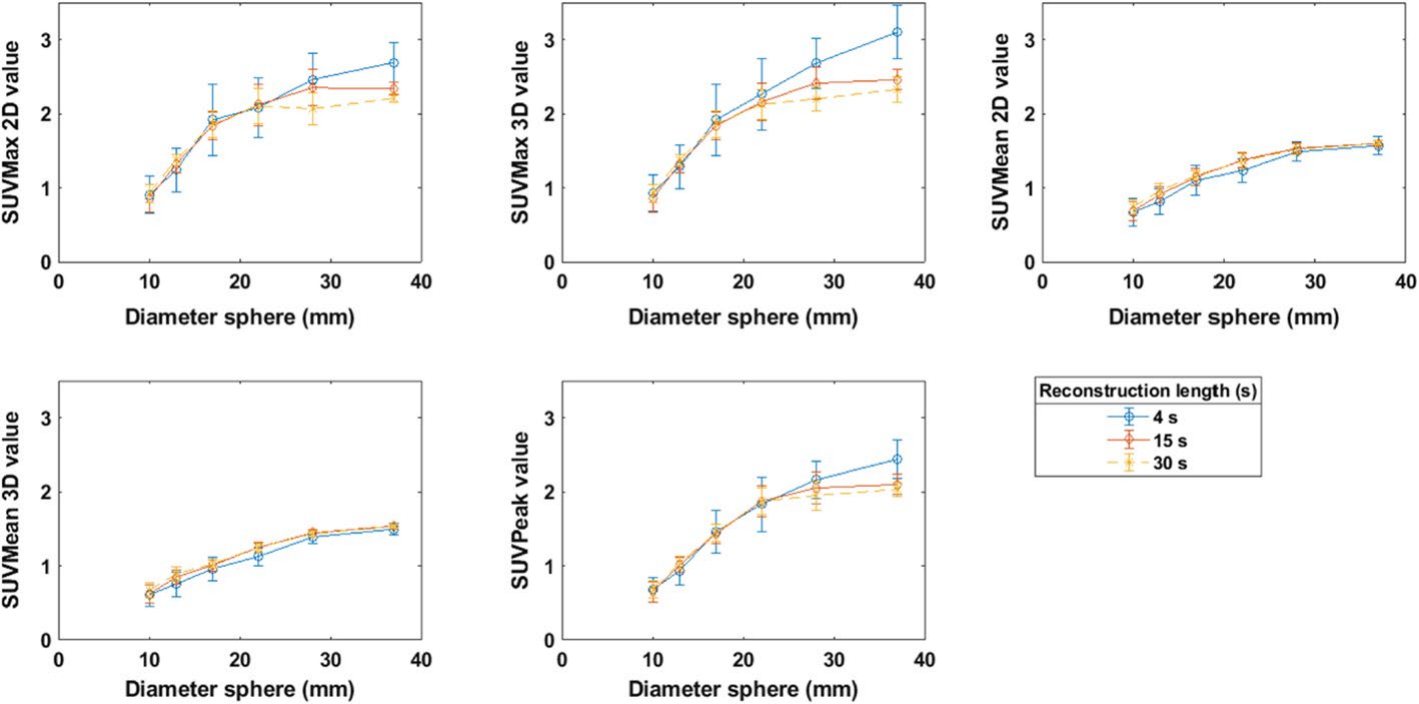
Average SUVMax 2D and 3D, SUVMean 2D and 3D and SUVPeak values (kBq/ml) and the corresponding standard deviation for spheres of different volumes for 4 s, 15 s and 30 s reconstruction lengths

After generating and reconstructing the images in the subsets with shorter reconstruction lengths, SUVMax 2D and 3D, SUVMean 2D and 3D and SUVPeak were calculated for the six spheres in each image of a subset. The resulting SUV metrics were averaged, and their standard deviation was estimated for each sphere. The average value was plotted as a function of the sphere diameter, obtaining a recovery curve based on SUVMax 2D and 3D, the SUVMean 2D and 3D and the SUVPeak. The results are shown in Fig. 1 as examples for the 4 s (blue line), 15 s (red line) and 30 s (yellow line) subsets.

For the larger spheres, for SUVMax and SUVPeak, the shorter reconstruction lengths tend to have a higher average SUV value than the longer reconstruction lengths, as shown for the three representative datasets (4 s, 15 s, 30 s reconstruction length) in Fig. 1.

**Fig. 2:**
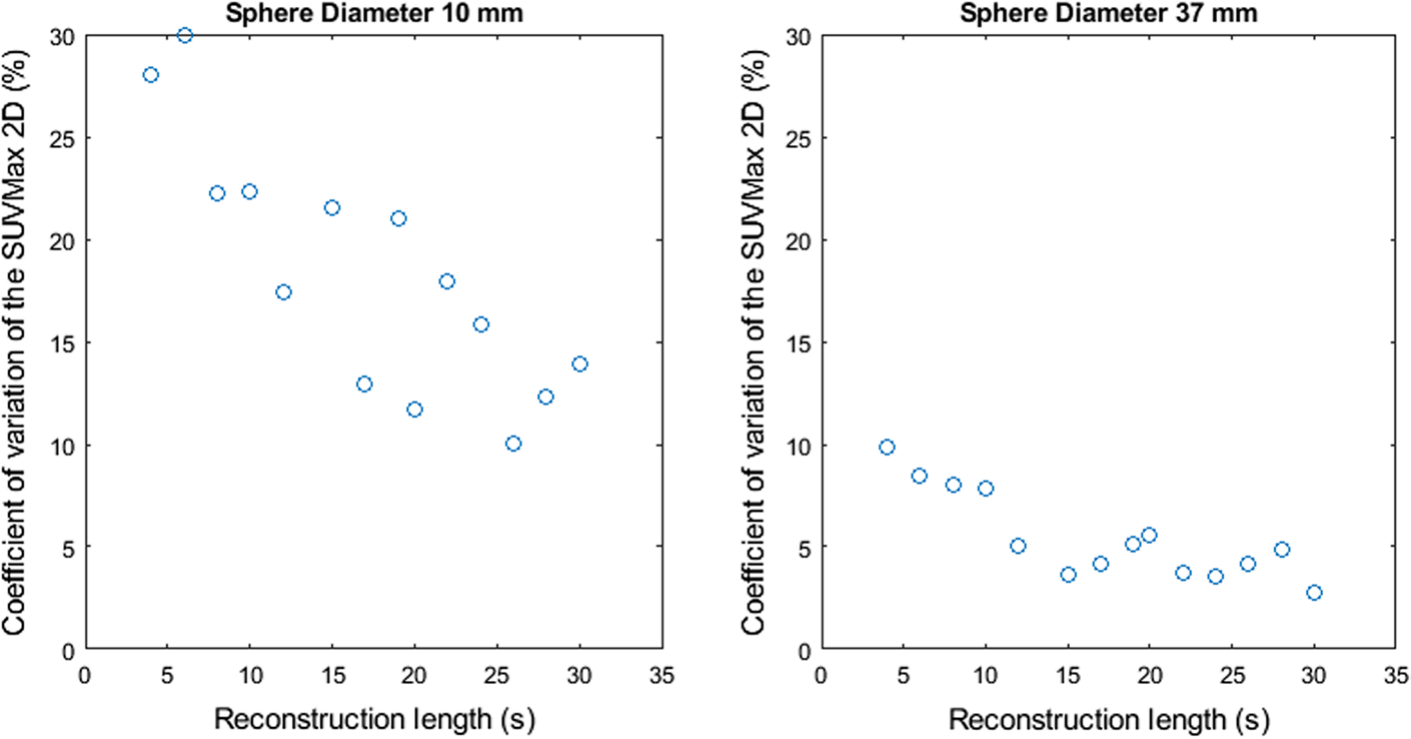
Coefficient of variation of SUVMax 2D as a function of the reconstruction length

**Fig. 3:**
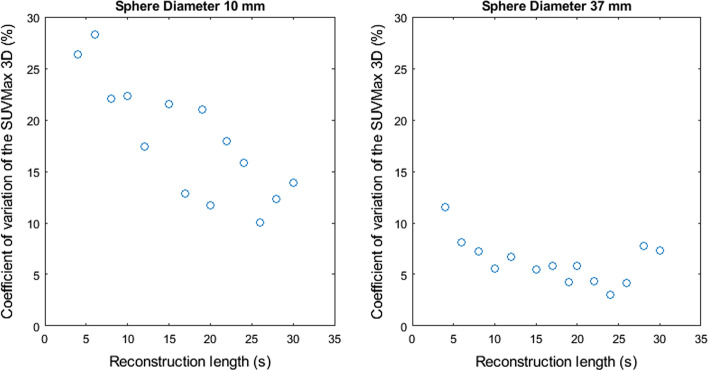
Coefficient of variation of SUVMax 3D as a function of the reconstruction length

**Fig. 4:**
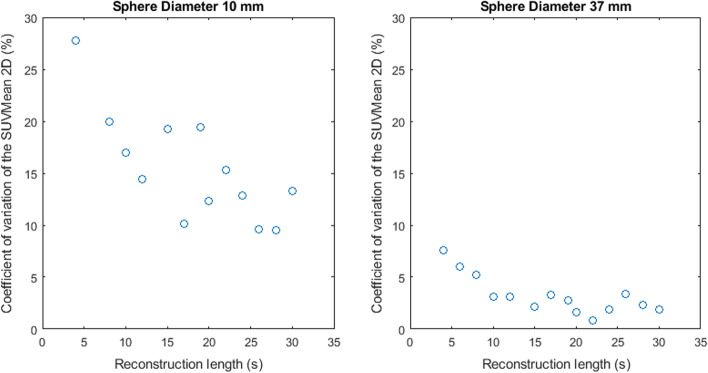
Coefficient of variation of SUVMean 2D as a function of the reconstruction length

**Fig. 5:**
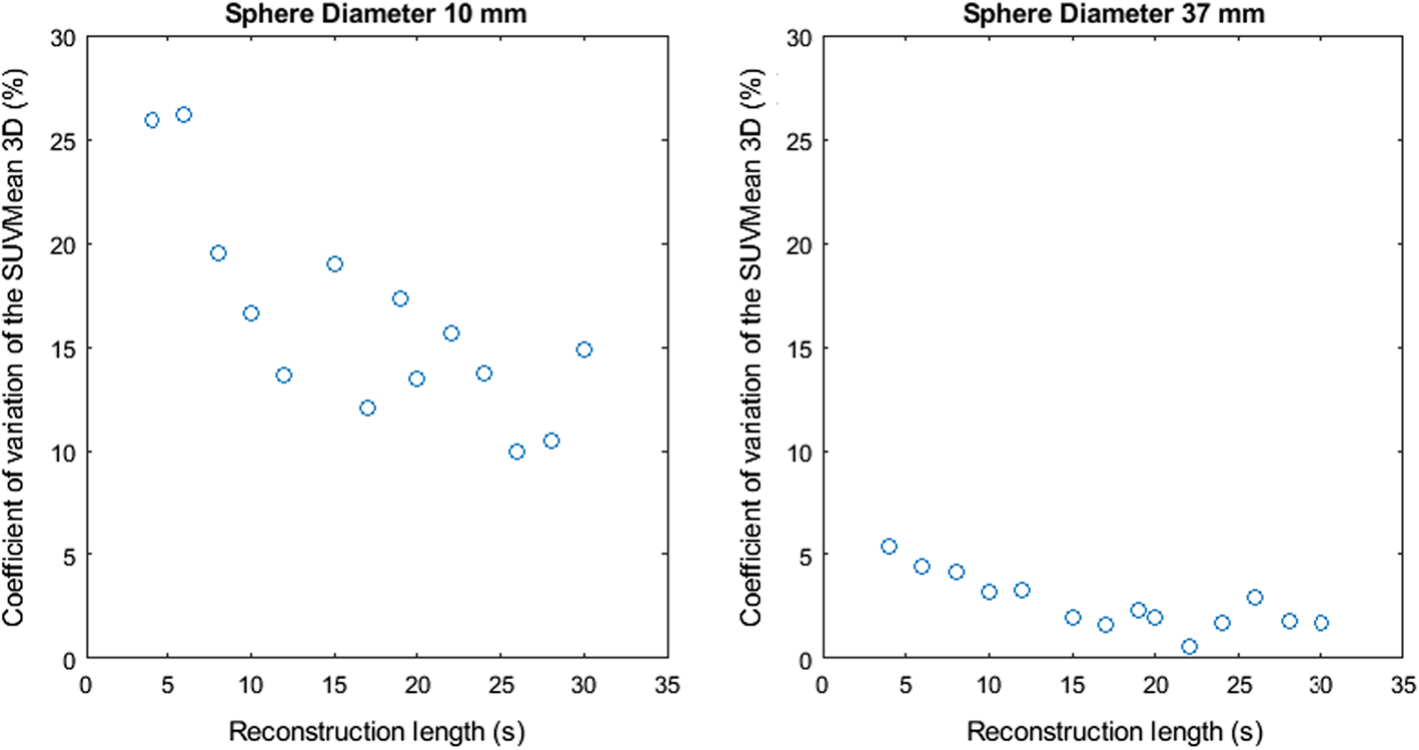
Coefficient of variation of SUVMean 3D as a function of the reconstruction length

**Fig. 6:**
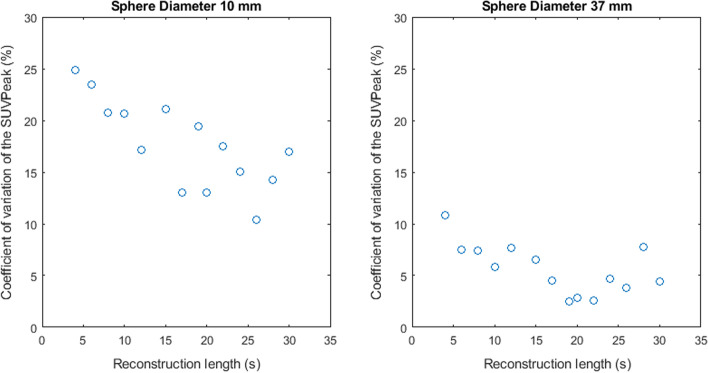
Coefficient of variation of SUVPeak as a function of the reconstruction length

Figures 2, 3, 4, 5 and 6 show the coefficient of variation of the measured SUV metrics. The coefficient of variation of the SUV is plotted as a function of the reconstruction length for the different spheres. We show the results for the spheres with 10 mm and 37 mm diameter as representative results.

**Fig. 7:**
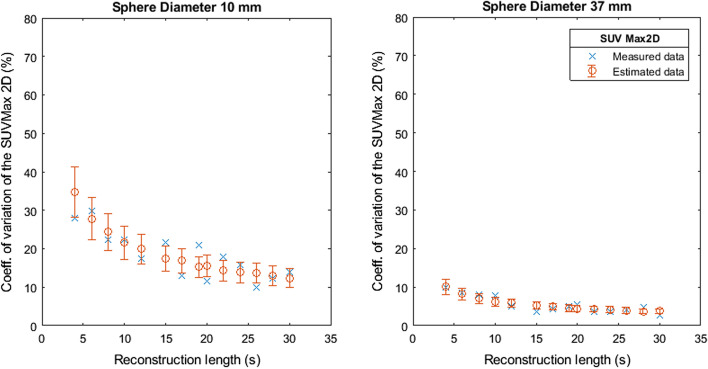
Measured and estimated coefficient of variation (mean and standard deviation) for SUVMax 2D as a function of the reconstruction length

**Fig. 8:**
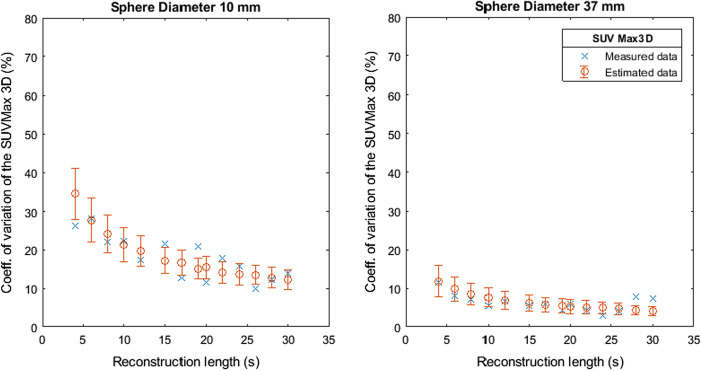
Measured and estimated coefficient of variation (mean and standard deviation) for SUVMax 3D as a function of the reconstruction length

**Fig. 9:**
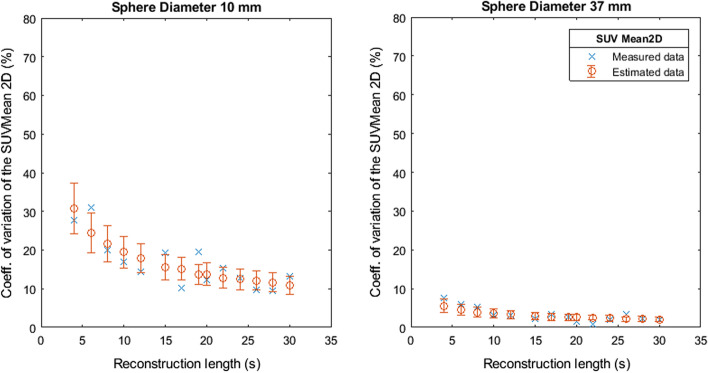
Measured and estimated coefficient of variation (mean and standard deviation) for SUVMean 2D as a function of the reconstruction length

**Fig. 10:**
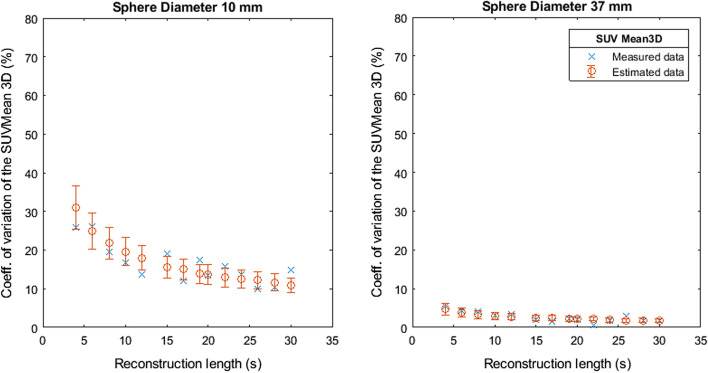
Measured and estimated coefficient of variation (mean and standard deviation) for SUVMean 3D as a function of the reconstruction length

**Fig. 11:**
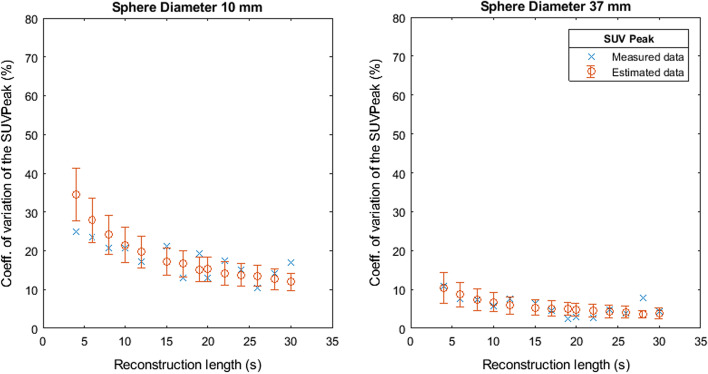
Measured and estimated coefficient of variation (mean and standard deviation) for SUVPeak as a function of the reconstruction length

Figures 7, 8, 9, 10 and 11 show the relation between the estimated and measured coefficients of variations. Figures 7, 8, 9, 10 and 11 show the measured coefficient of variation as reported in Figs. 2, 3, 4, 5 and 6 together with the estimated coefficient of variation (mean and standard deviation) obtained by using the coefficient of variation of the other reconstruction lengths as SD1 in Formula 2. We show the results for the smallest and largest sphere (10 mm and 37 mm diameter).

**Fig. 12:**
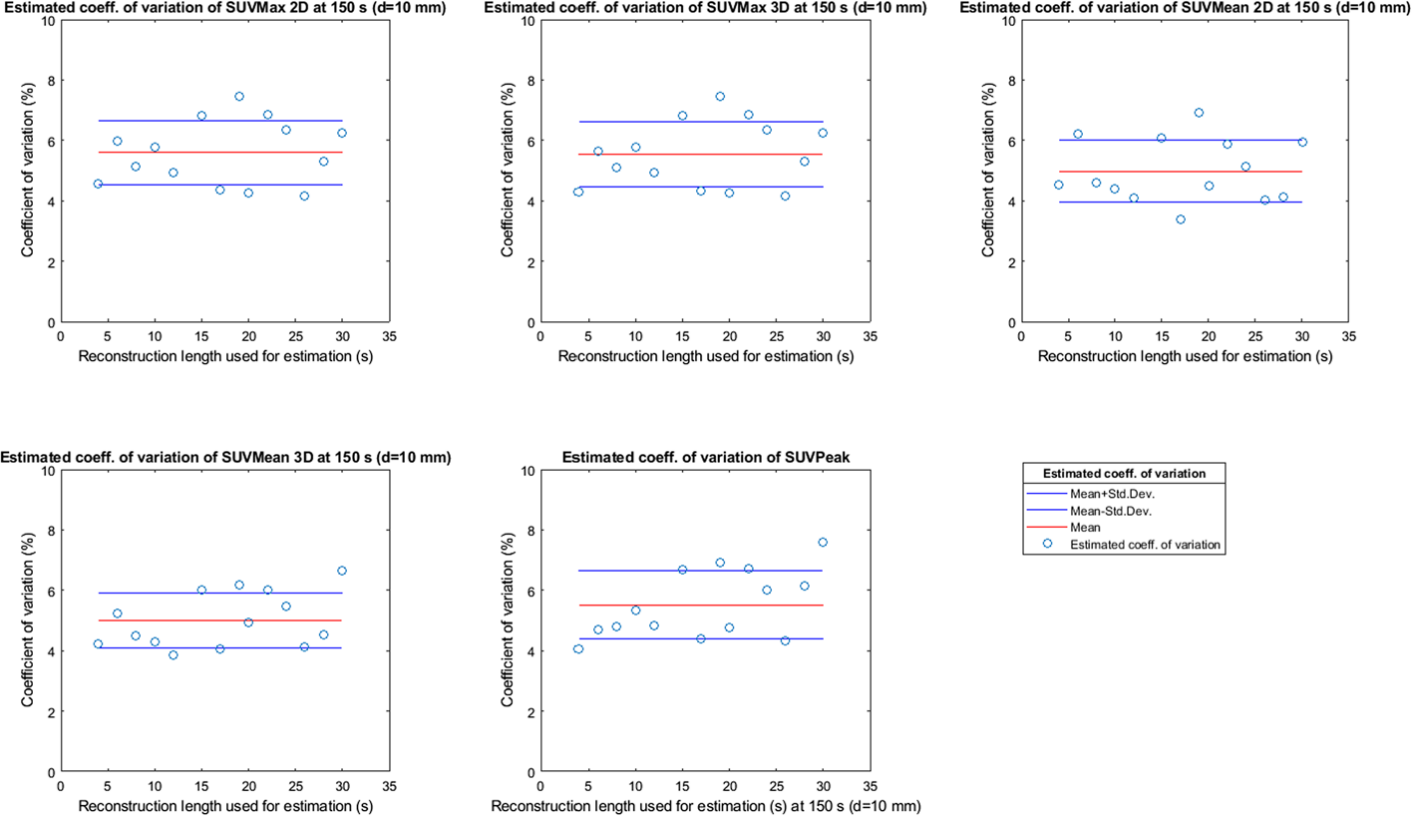
Estimated variation of SUV metrics for reconstruction length 150 s for a sphere of 10 mm

**Fig. 13:**
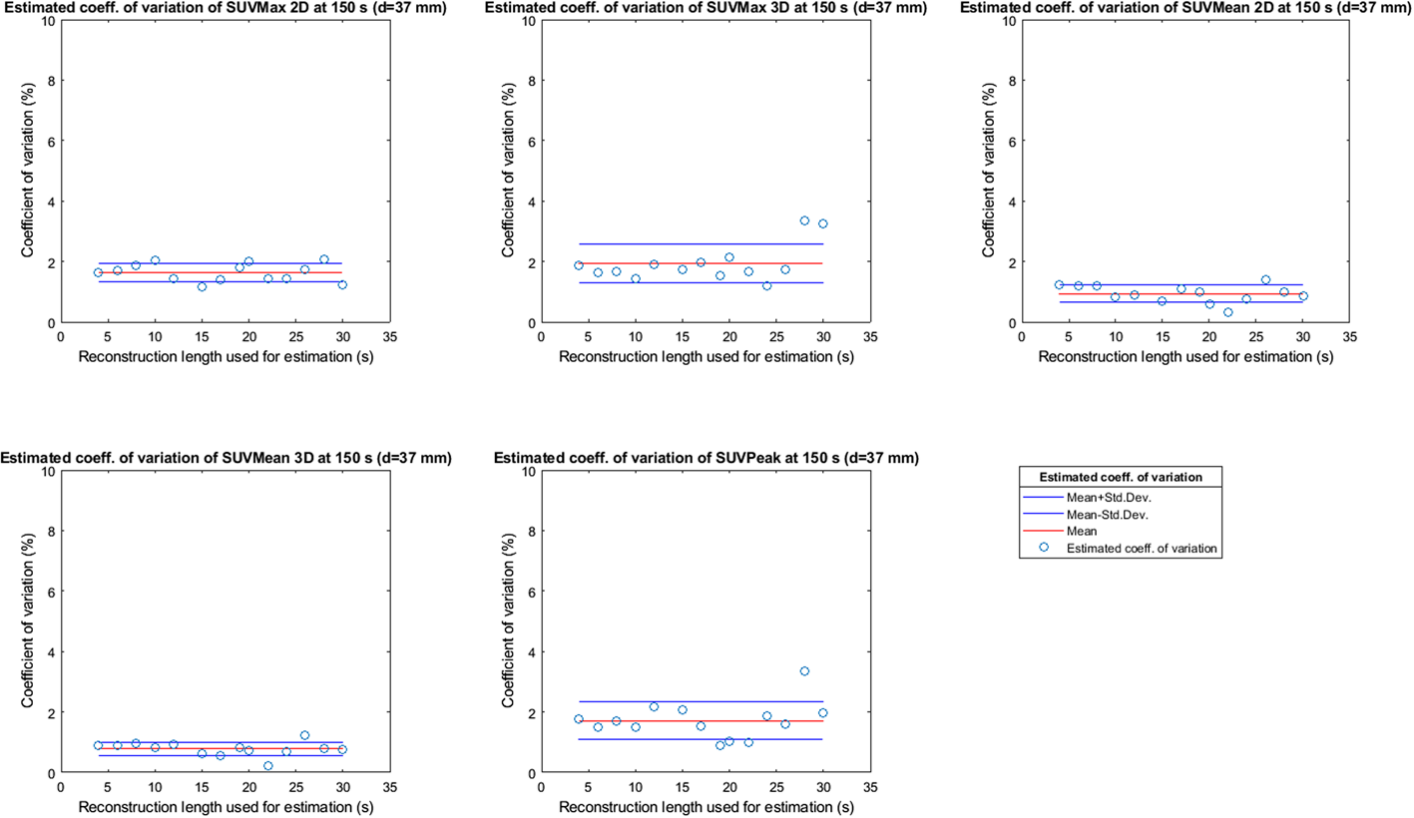
Estimated variation of SUV metrics for reconstruction length 150 s for a sphere of 37 mm

The results in Figs. 2, 3, 4, 5 and 6 were used to estimate the coefficient of variation of the 150 s original dataset according to Formula 2. The estimated variation of SUVMax 2D and 3D, SUVMean 2D and 3D and SUVPeak at 150 s reconstruction length is plotted in Fig. 12 for the 10-mm-diameter sphere and in Fig. 13 for the 37-mm-diameter sphere as examples. The dots show the estimated coefficient of variation of the SUV metrics at 150 s reconstruction length (SD2 in Formula 2) obtained by using the SUV metrics of the different subset as SD1 in Formula 2. Each figure also reports the average value (red line) plus and minus the standard deviation (blue lines) of the results, showing that the reconstruction length of the images in the subset has no structural effect on the estimated standard deviation at 150 s.

The estimated average coefficient of variation of SUVMax2D and 3D, SUVMean2D and 3D and SUVPeak at 150 s reconstruction length and its standard deviation, as shown, respectively, in red and blue in Figs. 12 and 13 for the 10 and 37 mm spheres, have been estimated for all the spheres and are summarized in Table 1. The data were statistically analysed to verify whether the difference between the estimated coefficient of variation of the SUV metrics was significant between spheres (same metric, different sphere diameter, so the difference between columns in Table 1) and between SUV metrics (different SUV metric, same sphere diameter, so the difference between rows in Table 1). The adjacent spheres with a significantly different coefficient of variation between them (*p* value t-test < 0.05) are underlined and in bold in Table 1.

**Table 1:** Average estimated coefficient of variation for SUV max, mean and peak for different spheres at 150 s reconstruction length

	*d*_sphere_ = 10 mm	*d*_sphere_ = 13 mm	*d*_sphere_ = 17 mm	*d*_sphere_ = 22 mm	*d*_sphere_ = 28 mm	*d*_sphere_ = 37 mm
Est. coeff. of Var SUVMax 2D	**5.6 ± 1.1%**	**3.8 ± 1.2%**	3.4 ± 0.8%	3.3 ± 0.7%	**2.9 ± 0.9%**	**1.6 ± 0.3%**
Est. coeff. of Var SUVMax 3D	**5.5 ± 1.1%**	**3.8 ± 1.2%**	3.4 ± 0.8%	3.1 ± 0.7%	**2.6 ± 0.6%**	**1.9 ± 0.6%**
Est. coeff. of Var SUVMean 2D	**5.0 ± 1.0%**	**3.7 ± 1.1%**	**2.8 ± 0.5%**	**2.2 ± 0.4%**	**1.7 ± 0.4%**	**0.9 ± 0.3%**
Est. coeff. of Var SUVMean 3D	**5.0 ± 0.9%**	**3.6 ± 0.9%**	**2.5 ± 0.6%**	**1.6 ± 0.3%**	**1.3 ± 0.3%**	**0.8 ± 0.2%**
Est. coeff. of Var SUVPeak	**5.5 ± 1.1%**	**3.6 ± 0.8%**	**2.8 ± 0.6%**	2.9 ± 0.7%	2.8 ± 0.8%	**1.7 ± 0.6%**

We report that the difference between the estimated coefficients of variation of the SUV metrics of the sphere with *d* = 10 mm and *d* = 13 mm and with *d* = 28 mm and *d* = 37 mm is significant for SUVMax, Mean and Peak. For SUVPeak, the difference between the estimated coefficients of variation of the 13 and 17 mm spheres is also significant. The difference between the estimated coefficients of variation of the SUVMean 2D and 3D is significant between each sphere.

Concerning the differences between SUV metrics, we report no significant difference between the estimated coefficient of variation of the SUV metrics of the two smaller spheres (*d* = 10, 13 mm). The values of SUVMean 3D are significantly lower than the values of SUVMax 2D, 3D and SUVPeak for the four larger spheres (*d* = 17, 22, 28, 37 mm). SUVPeak significantly differs from SUVMax 2D and 3D for *d* = 17 mm and from SUVMean 2D and 3D for *d* = 22, 28, 37 mm.

## Discussion

This study describes a method to estimate the statistical technical variability of SUV metrics and to compare the variability of SUV metrics between different lesion sizes. The proposed method determines the statistical technical variation of SUV metrics, which is a part of the total variation in PET imaging. The method's value lies in enabling the estimation of the influence of lesion size and choice of SUV metric on the total variation in a simple way.

In Fig. 1, the calculated values of the SUV metrics are shown for all spheres and for all reconstruction lengths. We report higher average SUV values for shorter reconstruction lengths. When the images are noisier, the chance is bigger than a single voxel or group of voxels will have a higher value due to a higher statistical variation. This effect occurs with SUVMax and SUVPeak, but not with SUVMean, where all the voxels in a region are used for calculation. The values in Fig. 1 could also be translated into recovery ratios in order to include the effect of background activity.

Figures 2, 3, 4, 5 and 6 show the coefficient of variation of the SUV metrics as a function of the reconstruction length for the different spheres. The higher variation at shorter reconstruction lengths reaches values up to 30% for the sphere with a 10 mm diameter. This suggests that when performing quantification of PET images on the small lesion and low counts, the effect of the statistical technical variability might not be negligible when compared with the variation used for diagnostic purposes. In this regard, the proposed method could be used to define the minimum required acquisition length: when the statistical technical variation of the SUV has become negligible to the test–retest variation, a longer acquisition time might not add value.

Figures 7, 8, 9, 10 and 11 show that the value of the measured and estimated coefficients of variation is comparable and that the standard deviation of the coefficients of variation is relatively small, indicating that this method can be applied to estimate the coefficient of variations at different reconstruction lengths. Figures 12 and 13 show that the choice of the reconstruction length of the subset used for the estimation is not creating a bias in the estimation of the coefficient of variation of the full-length dataset.

In Table 1, we report the result of the estimated standard deviation of the SUV metrics. We report significant differences in statistical technical variation for different sphere dimensions. The difference is always significant for each SUV metric for the smallest (diameter of 10 mm) and the largest (diameter of 37 mm) sphere. The difference is also significant between all spheres for the SUVMean. The coefficients of variation are typically ranging from 5% for the 10 mm sphere to 1% for the 37 mm sphere, in accordance with the range reported for simulated data [13]. In smaller spheres with lower recovery coefficients, we expect a larger influence of the noise on SUV metrics. Furthermore, for smaller spheres, a partial volume effect can introduce an extra source of variation in the quantification of SUV [14]. The maximum expected variation between images, for any estimated metric, did not exceed 6% for the smallest object (sphere of diameter 10 mm) and 2% for the largest object (sphere of diameter 37 mm) for a reconstruction length of 150 s. This provides an indication of the contribution of the statistical technical variation when the same scanner is used, with equal reconstruction length and activity, and can be compared with the variation measured in FDG PET test–retest studies reporting a typical variation of approximately 10% [15–17]. Our study is not a test–retest study, and it aims to quantify the variation obtained when (ideally) repeating the exact same acquisition, without changing any external factors, if not the statistical ones related to the characteristics of the emitters. The variation measured in our study is, therefore, smaller than the one typically measured in a test–retest study due to the fact that we do not have to deal with other factors such as repositioning of the patient or phantom and reinjection of the activity.

Nevertheless, it is important to notice that the value of the estimated statistical technical variation calculated for our scanner and reconstruction method is not directly translatable to other centres. The variability in the calculation of SUV metrics inhibits the direct comparison of these values [18]. Other factors introducing technical variability are, for example, acquisition settings, voxel size, reconstruction protocols, gating settings, analysis methods and scan duration, and their influence is too prominent for a direct comparison of the absolute values of the variation between scanners [7, 19].

For a given PET scanner, using advanced image reconstruction algorithms [20] will significantly improve the image quality in terms of noise and lesion detectability. Iterative PET reconstruction methods have been proven superior to filtered back projections (FBP) for their superiority in detecting focal regions and in reducing noise [21]. Furthermore, studies have shown that the noise correlation in FBP reconstruction might be object dependent and, therefore, it could not be possible to apply general statistical methods when estimating a coefficient of variation in different regions of interest in an image. [22] In our method, we have used an OSEM algorithm (an advanced Bayesian iterative reconstruction technique) because it is the usual choice when reconstructing whole-body F^18^ images. One of the advantages of an OSEM algorithm is that it aims to consider all the physical and statistical processes happening during data acquisition. On the other hand, Bayesian reconstruction algorithms penalize the formation of noisy images based on the hypothesis that large local variations in voxel intensity in the images are most likely due to noise. In OSEM algorithms, the degree of this penalization is unregulated, and the number of iterations is often reduced in order to control noise but at the cost of reducing contrast and lesion detectability [23]. Other reconstruction algorithms have shown better performances in noise reduction and lesion detectability. For example, point spread function (PSF) reconstruction algorithms can also be applied to PET images and have been shown to reduce noise and increase contrast in the reconstructed images identifying the potential for further reduction of the coefficient of variation in SUV metrics [24]. Furthermore, deep learning techniques have shown positive results in PET reconstruction applications [25], opening the possibility of reducing scan time or injected activity by up to 50% compared to OSEM algorithms [26]. Once again, we would like to underline that this paper only presents an example of the application of the method we describe for a specific Philips Gemini TF PET/CT system and its OSEM reconstruction algorithm.

The degree of statistical technical variation of an image is strongly dependent on imaging and reconstruction settings and needs to be evaluated for the specific scanner and algorithm in use. When evaluating the technical statistical coefficient of variation, standardization of the complete acquisition, from scanner and acquisition settings to PET reconstruction settings, is therefore strongly advised [7, 14, 27]. A simple method as the one described in this article can be routinely implemented to identify the contribution of the statistical technical variation in PET images. Once the degree of statistical technical variation is known, a user can evaluate its relevance to the total variation of SUV between clinical studies.

The SUV metrics (Max, Mean and Peak) present some significant differences for the same sphere diameter. For what concerns the smaller spheres (*d* = 10, 13 mm), the averaging step introduced in the calculation of SUVMean and Peak does not provide a significant difference in the coefficient of variation in our measurements. For larger lesions, the difference between the variation in SUV Mean 3D and SUV Peak is significant, suggesting that the dimension of the ROI used for averaging has a significant effect on SUV quantification and that a too large ROI might flatten the results. It is worth reminding that our definition of SUVMean was based on the knowledge of the measured objects, with a ROI defined as a sphere of diameter equal to the nominal diameter of the imaged sphere. This is not always possible during the analysis of images for diagnosis purposes. In that case, another definition of SUVMean must be used, and the variation between measurements might be expected to increase [12, 15, 16]. Furthermore, the biological factors present in clinical practice, such as glucose blood levels, rate of FDG uptake in the lesions or weight recording, can increase the SUV variation in diagnostic images [8, 28–31].

Another method to estimate the statistical technical variation would be to acquire a dataset with a longer acquisition time in comparison with the acquisition time used for diagnostic and generate subsets of the long acquisition with a time length similar to the one used for diagnostic. This could be a more direct way to measure the variation, possibly less susceptible to low photon statistics. A similar approach has been shown in [7] for SUVMax and Mean for reconstruction lengths of 5 min with variation between 11.2 and 1.2% depending on the filter, type of acquisition (2D or 3D) and metric (SUVMax or Mean) used. Another way to quantify voxel-based variation in SUV metrics has been reported in reference [32], showing higher sensitivity for SUV Mean (91.4%) than for SUV Max (82.0%).

For this study, we worked with a foreground-to-background activity ratio of 10:1. In order to verify the method further, it could be possible to repeat the evaluation with other ratios, for example, 5:1 and 2.5:1. Furthermore, the acquisitions could be repeated after a certain amount of hours in order to analyse the variation with other levels of noise. As previously discussed, a higher coefficient of variation can be expected for noisier images.

## Conclusion

In this study, we present a method to estimate the statistical technical variation of different SUV metrics. The method divides the total acquisition into subsets at different timeframes and estimates the expected coefficient of variation of the total acquisition using the standard deviation within the subsets. The method was tested on a 150-s acquisition with a foreground-to-background activity ratio of 10:1. This article shows how subsets of an original scan can be used to estimate the technical variation between images at different reconstruction lengths.

We used the method to estimate the statistical technical variation of SUV for different lesion sizes. The method shows that for our settings, the expected coefficient of variation of SUVMax, SUVMean and SUVPeak at a reconstruction length routinely used for clinical studies ranges between 5 and 6% for the smallest sphere (diameter of 10 mm) and between 0.9 and 2% for the largest sphere (diameter of 37 mm). These variations can be evaluated as relatively low when compared with the proposed PERCIST framework suggesting a 30% change in SUV as a significant variation of tumour activity [4]. The coefficient of variation reaches values up to 30% for shorter reconstruction lengths (in the order of 4 s), suggesting that the variation might become not negligible for noisier images with low counts. We also report different coefficients of variation for SUV Max, Mean and Peak. We, therefore, suggest including an evaluation of the statistical technical variation when defining the SUV metric of choice for clinical practice. Our method can be used routinely to provide insight into the statistical technical variation of a SUV quantification of images acquired with the same scanner and reconstruction method.

## Data Availability

The datasets used and/or analysed during the current study are available from the corresponding author.
